# Transperitoneal versus retroperitoneal approach does not alter arterial clamping strategy during robot-assisted partial nephrectomy: interim results from the multicentric PODRACING randomized controlled trial

**DOI:** 10.3389/fonc.2026.1767578

**Published:** 2026-03-16

**Authors:** Joris Vangeneugden, Saar Vermijs, Peter De Kuyper, Camille Berquin, Nicolaas Lumen, Victor Declerck, Frederic Baekelandt, Christophe Ghysel, Yannic Raskin, Bernard Bynens, Kenzo Mestdagh, Pieter De Backer, Pieter De Visschere, Charlotte Debbaut, Charles Van Praet, Karel Decaestecker

**Affiliations:** 1 Department of Urology, Ghent University Hospital, ERN eUROGEN Accredited Center, Ghent, Belgium; 2 Department of Human Structure and Repair, Faculty of Medicine and Health Sciences, Ghent University, Ghent, Belgium; 3 IBiTech-BioMMedA, Department of Electronics and Information Systems, Faculty of Engineering and Architecture, Ghent University, Ghent, Belgium; 4 Department of Urology, AZ Maria Middelares, Ghent, Belgium; 5 Department of Urology, AZ Sint-Jan Brugge, Bruges, Belgium; 6 Department of Urology, Ziekenhuis Oost-Limburg (ZOL) Genk, Genk, Belgium; 7 ORSI Academy, Melle, Belgium; 8 Department of Radiology and Nuclear Medicine, Ghent University Hospital, Ghent, Belgium

**Keywords:** 3D model, complications, main artery clamping, partial nephrectomy, randomized controlled trial, RAPN, retroperitoneal, selective clamping

## Abstract

**Introduction:**

Two anatomical approaches can be used to access a kidney tumor —and the associated renal hilum— during robot-assisted partial nephrectomy (RAPN): the transperitoneal (TP) and retroperitoneal (RP) approaches. Herein, we investigate whether the surgical approach has an impact on the performed clamping strategy: selective clamping (SC) vs main-artery clamping (MAC) and if a planned clamping strategy is performed equally accurate in both approaches. Furthermore, we look at total operative time and the occurrence of intraoperative and early postoperative complications in both approaches.

**Methods:**

Data from the interim analysis of the multicenter PODRACING randomized controlled trial (NCT06536439) was used, where patients undergo RAPN with or without a 3D perfusion zone (3DPZ) model. The study was approved by the Belgian Federal Agency for Medicines and Health Products (CIV-23-11-044854). TP surgery was performed with Da Vinci X or Xi systems, RP with Xi or SP systems (Intuitive Surgical, California, USA).

**Results:**

107 patients underwent RAPN in 4 participating Belgian centers from July 2024 until October 2025. In 56 cases, the surgery was performed TP, while in 51 cases a RP approach was used. The availability of a 3DPZ model did not differ across the different surgical approaches (χ2 p = 0.489). SC was performed in 73 (68%) cases and did not significantly differ between TP vs RP (χ2 p = 0.932). Also, planning and performing as planned a SC strategy (the primary endpoint of the PODRACING trial) did not significantly differ (χ2 p = 0.708). Median total operative time was significantly longer for TP (median 167 (IQR 140-210) min) than for RP (median 149 (IQR 113-186) min) (p = 0.024). Intraoperative and early postoperative complications were scarce in both groups.

**Conclusion:**

SC can be equally performed in the TP or RP approach, both in terms of frequency and accuracy of planning. Also, the occurrence of intraoperative and early postoperative complications did not differ between surgical approaches. This reassures us to further proceed with the PODRACING trial, allowing both TP and RP approaches, as no approach-related differences affecting the primary endpoint or short-term safety outcomes were detected in this interim analysis.

## 1 Introduction

Robot-assisted partial nephrectomy (RAPN) has become the standard of care for nephron-sparing surgery in localized renal tumors, offering oncological efficacy and preservation of renal function comparable to open and laparoscopic techniques (1, 2). Two principal anatomical approaches are employed: the transperitoneal (TP) and retroperitoneal (RP) routes. The choice between these approaches is influenced by tumor location (anterior vs posterior), size, complexity, patient body habitus, prior abdominal surgery, and surgeon expertise (3).

Comparative studies and meta-analyses demonstrate that the RP approach may be associated with shorter operative time, reduced estimated blood loss, and shorter hospital stay compared to the TP approach, while maintaining equivalent rates of positive surgical margins, major complications, and functional outcomes (3–5). The recent ROPARN randomized controlled trial further supports the feasibility and safety of the RP approach, particularly for dorsolateral tumors, with similar oncological outcomes and a trend toward reduced perioperative morbidity (6). However, the generalizability of these findings may be limited by center-specific expertise and patient selection.

Oncological outcomes, including positive surgical margin rates and recurrence, are comparable between TP and RP approaches across multiple studies, indicating that both techniques are oncologically sound when performed by experienced surgeons (5). Functional outcomes, such as preservation of estimated glomerular filtration rate (eGFR), are also similar, with tumor complexity and ischemia time often being the primary determinants of postoperative renal function.

Although debated, clamping strategy during RAPN, ranging from main-artery clamping (MAC) to selective clamping (SC) to no clamping, may also impact renal functional preservation (7). The choice of clamping strategy is tailored to tumor characteristics, vascular anatomy, and surgeon experience. SC, particularly when guided by three-dimensional perfusion zone (3DPZ) modeling, may spare postoperative renal function, especially in patients with impaired renal function or in complex tumours (8, 9). The benefits of using a 3DPZ model when planning and performing RAPN are currently being studied in the multicentric PODRACING randomized controlled trial (NCT06536439) (10). In the PODRACING trial, patients are randomized between using a 3DPZ or without. The primary endpoint entails planning and performing as planned a SC strategy. Secondary endpoints include all other functional and oncological outcomes. In this trial, surgeons are free to choose either a TP or RP approach. Since the renal artery and its branches are approached in a different fashion across both techniques, it might influence clamping strategy and thus potentially inflict a bias within the trial.

In the current study, using data from the interim analysis of the PODRACING trial, we therefore assess whether surgical access route (TP vs RP) is associated with -1- differences in clamping strategy employed (SC vs MAC), -2- adherence to the planned clamping strategy, -3- total operative time, and -4- incidence of intra-operative and early postoperative complications. These outcomes function as a first safety analysis of the PODRACING trial and to evaluate whether both approaches can be further used equivalently within the trial.

## 2 Methods

### 2.1 Data

This study utilized data from the interim analysis of the ongoing multicenter, prospective, randomized controlled PODRACING trial (NCT06536439). Patients with ≤3 ipsilateral renal masses cT1–2 planned for RAPN were randomized 1:1 between using a 3DPZ or not. Stratification was performed based on tumor complexity (PADUA < vs ≥10), the hospital where RAPN was performed and blinded assessment whether SC was deemed possible. The current interim analysis includes all consecutive patients enrolled between July 2024 and October 2025. The study protocol received ethical approval from the Belgian Federal Agency for Medicines and Health Products (reference: CIV-23-11-044854), and all participating centers complied with Good Clinical Practice guidelines. Written informed consent was obtained from every patient prior to inclusion.

### 2.2 Surgical procedures

All RAPN procedures were conducted by experienced robotic surgeons at participating institutions. TP RAPN was performed using either the *Da Vinci X* or *Da Vinci Xi* robotic platforms (Intuitive Surgical, Sunnyvale, CA, USA). RP RAPN was performed using the *Da Vinci Xi* or the *Da Vinci SP* system (also Intuitive Surgical, Sunnyvale, CA, USA), depending on institutional availability and surgeon preference.

Tumor resection, renorrhaphy techniques, and postoperative management strategies adhered to each center’s standardized RAPN protocols. Clamping approach, SC or MAC, was determined by the surgeon preoperatively based on preoperative imaging with or without 3DPZ modeling and was documented before surgery commenced.

### 2.3 Endpoints and definitions

The primary variable of interest for this analysis was the execution of the intended clamping strategy, defined as whether the preoperatively planned SC was successfully performed intraoperatively (primary endpoint of the PODRACING trial). Secondary variables in this analysis include the type of clamping strategy actually performed (SC vs MAC) and the occurrence of intraoperative complications. Complications were defined according to intraoperative clinical documentation and classified descriptively (not following specific guidelines) (ref ICARUS DOI: 10.1016/j.euf.2022.01.018). Blood loss, postoperative kidney function, detailed operative times, long-term postoperative complications and other measures are herein considered outcomes rather than safety matters and will be analyzed in future research.

### 2.4 Statistical analysis

Associations between surgical approach (TP vs RP) and categorical variables, including concordance between planned and performed SC and also performed clamping technique (SC vs MAC), were evaluated using a Chi-squared (χ²) test. Statistical significance thresholds were predefined according to the main study protocol and set at *p* < 0.05. Total operative time (TP vs RP) was assessed using an independent two-sample t-test. Intraoperative and early postoperative complications were summarized using descriptive statistics. No imputation was performed for missing data given the interim nature of this analysis.

## 3 Results

A total of 107 patients were included in this interim analysis, all of whom underwent RAPN across four Belgian centers between July 2024 and October 2025. Patient characteristics can be found in **Table 1**. Of these procedures, 56 (52%) were performed via a TP approach, while 51 (48%) utilized a RP access route. The number of performed approaches per center is displayed in **Table 2**. TP was performed in more than 90% of patients in center 1 and center 3, but only in 12% in center 2 and in 23% in center 4. The distribution of patients receiving a 3DPZ model did not differ significantly between the TP and RP groups (χ², *p* = 0.489) (**Table 3**), indicating comparable allocation across surgical approaches.

**Table 1:** Patient characteristics of the studied interim cohort.

Characteristic	Value
Sex, n (%)	
M	65 (61%)
F	42 (39%)
Age at surgery, median (IQR)	65 (59-75) years
ASA score, median (IQR)	2 (2-3)
CCI score, median (IQR)	5 (4-6)
Tumor diameter, median (IQR)	31 (22-41) mm
Preoperative biopsy, n (%)	2 (2%)
RENAL score, median (IQR)	7 (6-8)
Tumor location, n (%)	
Anterior (a)	47 (44%)
Posterior (p)	40 (37%)
Not differentiable between a or p (x)	20 (19%)
PADUA score, median (IQR)	8 (7-9)
Number of ipsilateral tumors treated, n (%)	
1	103 (96%)
2	4 (4%)
Preoperative creatinine, median (IQR)	0.92 (0.78-1.05) mg/dl
Preoperative eGFR, median (IQR)	80 (61-90) ml/min

**Table 2:** Number (%) of performed surgical technique per center (anonymous).

	TP	RP
Center 1	33 (92%)	3 (8%)
Center 2	5 (12%)	37 (88%)
Center 3	15 (94%)	1 (6%)
Center 4	3 (23%)	10 (77%)

**Table 3:** Distribution across TP vs RP of patients receiving a 3DPZ model (χ², p = 0.489).

	3DPZ model available	No 3DPZ model	Total
TP	29 (27%)	27 (25%)	56 (52%)
RP	23 (21%)	28 (26%)	51 (48%)
Total	52 (49%)	55 (51%)	107 (100%)

Regarding vascular control, SC was used in 73 cases (68%), whereas in 34 cases (32%) MAC was used. The frequency of SC did not significantly vary between TP and RP procedures (68% vs 69%, χ² *p* = 0.932) (**Table 4**). Similarly, the ability to execute SC according to the preoperative plan showed no significant association with surgical access route (64% TP vs 61% RP, χ² *p* = 0.708) (**Table 5**).

**Table 4:** Distribution across TP vs RP of performed clamping strategy (SC vs MAC) (χ², p = 0.932).

	SC	MAC	Total
TP	38 (36%)	18 (17%)	56 (52%)
RP	35 (33%)	16 (15%)	51 (48%)
Total	73 (68%)	34 (32%)	107 (100%)

**Table 5:** Distribution across TP vs RP for planning and performing as planned as SC strategy (primary endpoint of the PODRACING trial) (χ², p = 0.708).

	SC performed as planned	SC not performed as planned or no SC	Total
TP	36 (34%)	20 (19%)	56 (52%)
RP	31 (29%)	20 (19%)	51 (48%)
Total	67 (63%)	40 (37%)	107 (100%)

**Figure 1:**
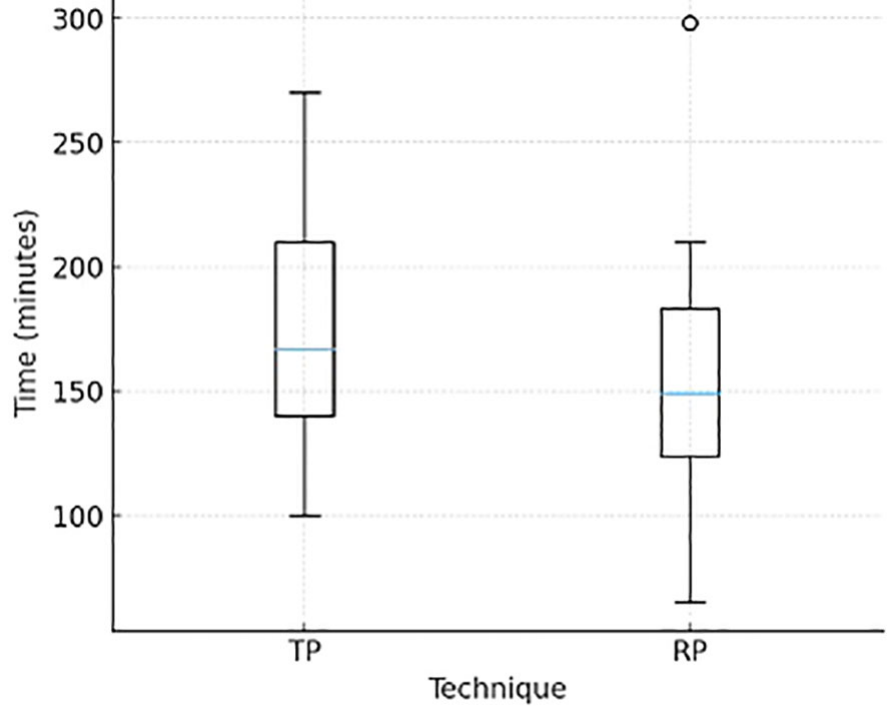
Boxplot displaying total operative time (min) (TP vs RP). Boxes represent the interquartile range with median values indicated, shown to illustrate the distribution of operative time; statistical comparisons were performed using mean ± SD.

Mean total operative time was significantly longer for TP compared with RP (174.6 ± 44.6 min vs. 151.0 ± 47.9 min; *p* = 0.024). Distribution of total operative time is displayed in **Figure 1**.

Intraoperative complications were uncommon overall, occurring in six patients (5.6%), and are summarized in **Table 6**.

**Table 6:** Summary of reported intraoperative complications during TP or RP RAPN.

	Intraoperative complication
TP (n=4)	- Reported excessive bleeding - Rupture of a small arterial aneurysm on a selective branch after clamping, leading to clipping of this branch - Small iatrogenic liver lesion requiring hemostatic agent - Small and serosal iatrogenic bowel tear requiring suturing
RP (n=2)	- Reported excessive bleeding - Conversion to radical nephrectomy (because of unexpected venous tumor thrombus)

Early postoperative complications were also scarce, occurring in four patients (3.7%) during the hospitalization course and two patients (1.9%) in the first month of follow-up (**Table 7**) and are all classified as Clavien-Dindo grade 1 or 2.

**Table 7:** Summary of reported early postoperative complications during TP or RP RAPN (and associated Clavien-Dindo (CD) grade).

	Complication during hospitalization		Complication during first postoperative month
TP (n=3)	- 2x urinary retention requiring catheter (CD 1) - Pneumonia requiring antibiotics (CD 2)	TP(n=1)	- Wound infection requiring topical antiseptic treatment (CD 1)
RP (n=3)	- Urinary retention requiring catheter (CD 1) - Paresthesia of the right radial nerve (CD 1) - Retroperitoneal hematoma and associated pyelonephritis requiring blood transfusion and antibiotics (CD 2)	RP(n=1)	- Constipation (conservative) (CD 1)

## 4 Discussion

In this interim analysis of the PODRACING randomized controlled trial, we evaluated whether the choice of surgical access route, TP vs RP, influenced the feasibility of performing SC and the adherence to the planned clamping strategy, total operative time, or the occurrence of intraoperative and early postoperative complications during RAPN. Despite the substantial anatomical differences between both approaches and a longer operative time in the transperitoneal approach, our results demonstrate no significant differences in the frequency of SC, accuracy in executing SC as preoperatively planned, or complication rates. These findings carry important implications for the ongoing PODRACING trial, particularly regarding the robustness of its primary endpoint across different surgical techniques. It should be emphasized that the observed preference for a TP or RP approach largely reflects established institutional practice within the participating centers and represents a contextual characteristic of this interim cohort rather than a methodological limitation requiring adjustment.

There is no direct evidence in the literature comparing SC vs MAC strategies specifically between TP and RP approaches. Existing comparative studies and meta-analyses of clamping strategies do not stratify outcomes by surgical approach (11). Most available data focus on the impact of clamping technique on renal function preservation, perioperative outcomes, and feasibility, but do not address whether the benefits or risks of selective clamping differ by approach. We herein present thus the first piece of evidence regarding this topic.

Several factors influence a surgeon’s choice for RP or TP access. First of all, the training and experience of the surgeon. With the TP approach, as long as the intraperitoneal cavity is accessible, all possible tumors can be accessed. This is seen in two centers in our trial that perform TP RAPN in almost all patients (center 1 and center 3, 92% and 94% respectively), whereas in the other two centers, the TP approach is used only in a minority (center 2 and center 4, in 12% and 23%, respectively). Secondly, the location of the tumor. The RP approach may be challenging when accessing tumors on the upper pole and close to the hilum, as well as very large tumors. However, RP approach excels in terms of shorter operative time, especially for posterior tumors, for which the TP approach mandates dissection of almost the entire kidney to flip it and gain access to the tumor (3). Access to different robotic platforms plays a key role. With the Da Vinci SP, available in two of the participating centers (center 2 and 3) the supine anterior retroperitoneal access (SARA) allows for RP surgery while the patient is in supine position. This avoids lateral decubitus, which is mandatory for TP kidney surgery. In the end, the choice for TP or RP is based on a combination of all these factors.

Regarding intraoperative complications, our complication rate of 5.6% aligns with previously reported rates in large RAPN series. Tanagho et al., for example, reported a 6% intraoperative complication rate in 886 RAPN procedures, remarkably similar to our interim findings (12). Postoperative complications also align with existing literature and are low in the era of minimally-invasive surgery and when performed by expert hands (3–5). Importantly, given the small occurrence, no meaningful difference in complication type or frequency was observed between TP and RP access in our study, which mirrors the findings of multiple comparative analyses (3, 5, 13). RP access is sometimes described as providing faster and more direct access to the renal hilum with less bowel manipulation, potentially translating into reduced approach-related complications. This is also demonstrated in our study, where in the TP group minor liver and bowel lesions are noted. Our study demonstrates that performing RP RAPN is significantly faster than TP RAPN, as previously shown in multiple other studies (3, 6). As stated before, the main reason for this entails the faster access to the renal hilum with less considerable organs delaying dissection. However, this should be further studied using detailed/sub-divided operative times in the final analysis of the RCT.

Our findings provide early reassurance that the primary endpoint of the PODRACING trial —the accuracy of performing SC as planned— is robust across both surgical approaches. This is relevant because SC relies on clear understanding of hilar vasculature, which is often more readily visualized in the TP approach due to greater working space, while the RP approach offers direct but more narrow access to the hilum. Despite these conceptual differences, our data indicate that surgeons can achieve similar vascular control outcomes through either route, reinforcing the feasibility of allowing approach choice to remain unstandardized within the trial.

### 4.1 Limitations

This interpretation of interim results of a randomized trial is not free of limitations. First, the analysis reflects an early, though predefined, subset of the total planned sample size, which may limit statistical power. Second, although the study is multicentric, variations in surgeon experience, robotic platform availability, and institutional preference for TP versus RP access may introduce center-related bias. We do not account for tumor-related factors such as tumor location. However, when looking at these data, all kinds of tumors are approached by different approaches or robotic systems (e.g. both anterior and posterior, and both surgically less or more complex tumors are treated with the SP robot, or are treated by surgeons only performing TP). We did not record the exact reason why a surgeon opted for RP or TP access. Further analysis of tumor and patient-related influence (patient comorbidities, tumor complexity, tumor location etc.) will follow in the main RCT reports. A third limitation entails that the interim design precludes assessment of longer-term postoperative and functional outcomes, meaning that conclusions are currently limited to short-term performance and safety. These limitations highlight the need for cautious interpretation until final trial results become available. The final analysis will incorporate additional perioperative and postoperative endpoints, including detailed operative times, warm ischemia duration, postoperative complication rates, renal functional outcomes, and other patient- and surgeon-related outcomes. Together, these data will provide a more comprehensive evaluation of whether 3DPZ modeling influences surgical decision-making, operative safety, and patient outcomes across both TP and RP RAPN.

## 5 Conclusion

Both the TP and RP approaches allowed surgeons to execute SC or MAC at similar frequencies, and the concordance between the preoperative plan and intraoperative clamping strategy was equally high in each access route. Furthermore, no differences were observed in the rate or nature of intraoperative or early postoperative complications. This suggests that both TP and RP RAPN can be regarded as comparably safe and effective during the vascular control phase of surgery, and reassures us to further proceed with the PODRACING trial, applying both TP or RP approaches, as no approach-related differences affecting the primary endpoint or short-term safety outcomes were detected in this interim analysis.

## Data Availability

The raw data supporting the conclusions of this article will be made available by the authors, without undue reservation.
