# Correction: Transperitoneal versus retroperitoneal approach does not alter arterial clamping strategy during robot-assisted partial nephrectomy: interim results from the multicentric PODRACING randomized controlled trial

**DOI:** 10.3389/fonc.2026.1927322

**Published:** 2026-07-17

**Authors:** Joris Vangeneugden, Saar Vermijs, Peter De Kuyper, Camille Berquin, Nicolaas Lumen, Victor Declerck, Frederic Baekelandt, Christophe Ghysel, Yannic Raskin, Bernard Bynens, Kenzo Mestdagh, Pieter De Backer, Pieter De Visschere, Charlotte Debbaut, Charles Van Praet, Karel Decaestecker

**Affiliations:** 1 Department of Urology, Ghent University Hospital, ERN eUROGEN Accredited Center, Ghent, Belgium; 2 Department of Human Structure and Repair, Faculty of Medicine and Health Sciences, Ghent University, Ghent, Belgium; 3 IBiTech-BioMMedA, Department of Electronics and Information Systems, Faculty of Engineering and Architecture, Ghent University, Ghent, Belgium; 4 Department of Urology, AZ Maria Middelares, Ghent, Belgium; 5 Department of Urology, AZ Sint-Jan Brugge, Bruges, Belgium; 6 Department of Urology, Ziekenhuis Oost-Limburg (ZOL) Genk, Genk, Belgium; 7 ORSI Academy, Melle, Belgium; 8 Department of Radiology and Nuclear Medicine, Ghent University Hospital, Ghent, Belgium

**Keywords:** 3D model, complications, main artery clamping, partial nephrectomy, randomized controlled trial, RAPN, retroperitoneal, selective clamping

The **Conflict of interest** statement was erroneously given as “The author(s) declared that this work was conducted in the absence of any commercial or financial relationships that could be construed as a potential conflict of interest.”

The correct **Conflict of interest** statement is “Author PDB has been a co-founder and shareholder of MEDANNOT s.r.o. (Spania Dolina, Slovakia) since March 2024. MEDANNOT is an online platform for segmentation of CT scans into 3D models. The remaining authors declare that this work was conducted in the absence of any commercial or financial relationships that could be construed as a potential conflict of interest.”

The original version of this article has been updated.

